# Diversity of Nonribosomal Peptide Synthetase Genes in the Microbial Metagenomes of Marine Sponges

**DOI:** 10.3390/md10061192

**Published:** 2012-05-25

**Authors:** Sheila Marie Pimentel-Elardo, Lubomir Grozdanov, Sebastian Proksch, Ute Hentschel

**Affiliations:** 1 Department of Biochemistry and Biomedical Sciences, McMaster University, 1280 Main St.W, Hamilton, ON L8S 4K1, Canada; Email: elardos@univmail.cis.mcmaster.ca; 2 Julius-von-Sachs Institute for Biological Sciences, University of Würzburg, Julius-von-Sachs Platz 3, 97082 Würzburg, Germany; Email: lubomir.grozdanov@gmail.com (L.G.); basti.proksch@googlemail.com (S.P.)

**Keywords:** nonribosomal peptide synthetase, NRPS, marine sponge, Porifera, metagenomics

## Abstract

Genomic mining revealed one major nonribosomal peptide synthetase (NRPS) phylogenetic cluster in 12 marine sponge species, one ascidian, an actinobacterial isolate and seawater. Phylogenetic analysis predicts its taxonomic affiliation to the actinomycetes and hydroxy-phenyl-glycine as a likely substrate. Additionally, a phylogenetically distinct NRPS gene cluster was discovered in the microbial metagenome of the sponge *Aplysina aerophoba*, which shows highest similarities to NRPS genes that were previously assigned, by ways of single cell genomics, to a *Chloroflexi* sponge symbiont. Genomic mining studies such as the one presented here for NRPS genes, contribute to on-going efforts to characterize the genomic potential of sponge-associated microbiota for secondary metabolite biosynthesis.

## 1. Introduction

Sponges (phylum Porifera) are an extraordinarily rich source for bioactive metabolites [1]. Current hypothesis holds that because sponges lack physical defenses, they are exposed to an enormous predator and epibiont pressure, which, in turn, has provoked the evolution of structurally highly diverse, effective and sophisticated chemical defenses. Many sponges also contain massive amounts of microorganisms extracellularly within the mesohyl matrix, which may constitute up to one third of the animal’s biomass [2]. There is increasing evidence that important marine natural product classes, complex polyketides and nonribosomal peptides, are truly synthesized by symbiotic bacteria rather than by the sponge itself [3]. However, since the vast majority of sponge symbionts, much like most environmental bacteria, are still refractory to cultivation, new experimental approaches are needed to provide information about their genomic potential for secondary metabolite biosynthesis. Methods such as metagenomics, and more recently single-cell genomics were developed to access the DNA pool of complex environmental microbial consortia in a cultivation-independent manner [4]. 

Nonribosomal peptide synthetases (NRPS) are large, multimodular enzymes that are organized in modules containing specific domains that sequentially incorporate amino acid building blocks into a growing peptide chain [5,6]. A typical NRPS module contains an adenylation (A) domain, a peptidyl carrier protein domain and a condensation domain. A thioesterase domain frequently terminates chain elongation. NRPS gene clusters encode for a wide range of nonribosomal peptides, ranging from antibiotics (e.g., penicillin, vancomycin [7]), toxins (e.g., kahalalide F [8]), siderophores (enterobactin, vibriobactin [9]) to anti-inflammatorials and immunosuppressants (e.g., cyclosporin A [10]). These pharmacologically relevant bioactivities have motivated extensive searches for novel NRPS genes in microbial isolates and in environmental samples. For example, degenerate oligonucleotide primers that target the conserved region of the A domain have been used to unravel the diversity of NRPS genes in actinobacterial and fungal endophytes of plants [11,12], in actinobacterial [13,14,15] and fungal [16] isolates of marine sponges, and in free-living freshwater cyanobacteria [17], and marine actinobacteria [18,19]. Mixed PKS-NRPS gene clusters from the marine sponge *Discodermia dissoluta* were furthermore reported using a metagenomic approach [20] and a bimodular NRPS gene cluster was cloned from a *Chloroflexi* symbiont of the marine sponge *Aplysina aerophoba* by phi29-mediated whole genome amplification [21]. 

The aim of the current study was to explore the presence and diversity of NRPS genes in the microbial metagenomes of marine sponges. PCR screening of diverse marine sponge species and of metagenomic libraries, followed by cloning of a NRPS gene cluster, were employed towards this goal. This study represents a continuation of previous efforts where the diversity, distribution and genomic context of gene clusters relevant for secondary metabolism, such as polyketide synthases [22,23] and halogenases [24], were investigated in sponge associated microbial consortia.

## 2. Results and Discussion

### 2.1. NRPS Gene Diversity

**Figure 1:**
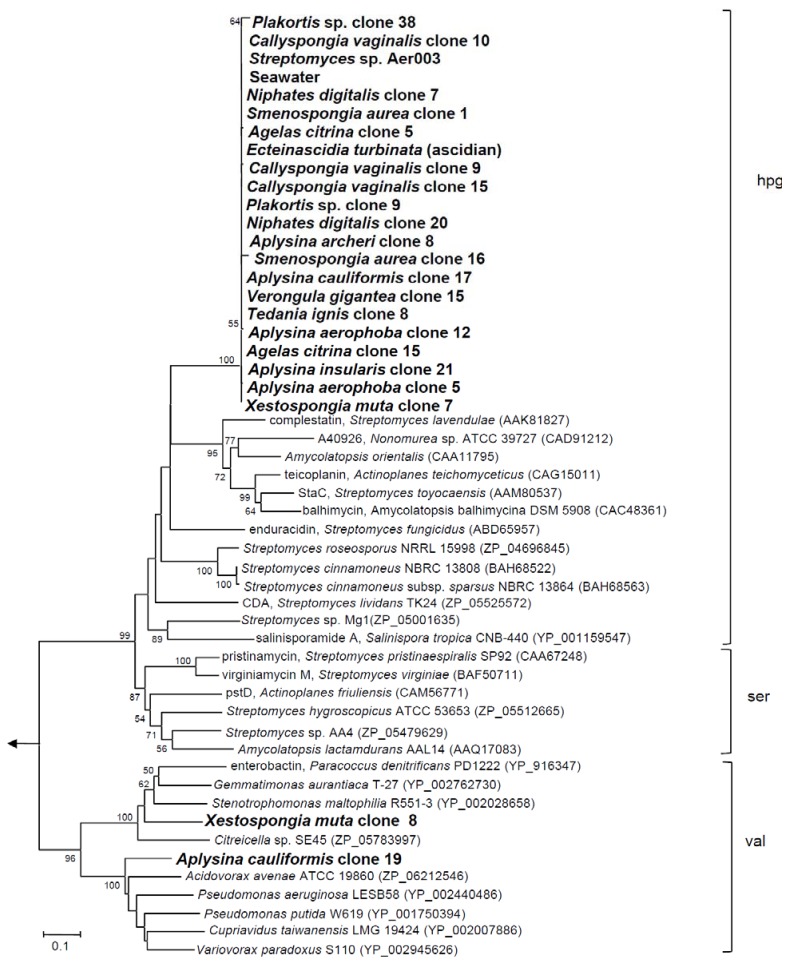
Neighbor-joining tree of translated nonribosomal peptide synthetase (NRPS) A domains. Bootstrap values greater than 50% are at the nodes. The arrow points to the archaeal outgroup, *Methanothermobacter thermoautotrophicus* (NP_275799). The scale bar indicates 0.1 substitutions per nucleotide position. Substrate specificities correspond to 4-hydroxy-phenyl-glycine (hpg), serine (ser), and valine (val). Phylogenetic trees were constructed using MEGA version 4 with Poisson correction model for amino acids, complete deletion of gaps and bootstrap consisting of 1000 replications [27].

We first aimed to investigate the NRPS gene diversity in twelve marine sponge species from the Bahamas and the Mediterranean. For this purpose NRPS A domain gene fragments were amplified as previously described [25] using degenerate primers A3 and A7R. Altogether 62 A domain DNA sequences (*ca*. 750 bp) were amplified from the metagenomes of all 12 sponge species, from the ascidian *Ecteinascidia turbinata* and from seawater. No PCR product was obtained from the sediment sample. Additionally, a NRPS A domain from the bacterium *Streptomyces* sp. Aer003, which had previously been isolated from Mediterranean *Aplysina aerophoba* (Acc# JN830622) was amplified and sequenced. Sequences from each sponge metagenome that exhibited ≥98% sequence identities were presumed to have been amplified from identical genes, thus taking into account the PCR-induced errors that may arise from using degenerate primers [20]. Using this criterion, a total of 24 sequences were considered different (Acc# JN815085–JN815111). A neighbor-joining tree was constructed including related sequences from BLAST analysis (Figure 1). 

The majority of these sequences (22/24) formed one large distinct cluster with 97–99% in-cluster amino acid identity. The cluster also included the NRPS A domain sequences from the isolate *Streptomyces* sp. Aer003 and from seawater. The closest relatives were all *Actinobacteria* with the A domain sequences from *Streptomyces roseosporus* (ZP_04696845) and *S. fungicidus* (ABD65957) being the closest relatives (64, 65% sequence identity). The substrate for the NRPS adenylation domains appears to be hydoxy-phenyl-glycine (hpg) as predicted by NRPSpredictor2 [26]. Notably, *Xestospongia muta* clone 8 and *Aplysina cauliformis* clone 19 fell outside of this large cluster with the A domain sequences of *Stenotrophomonas maltophila* (YP_002028658) and *Pseudomonas putida* (YP_001750394) being their closest phylogenetic neighbors, respectively and with valine being the likely substrate. Overall, the NRPS gene diversity discovered in this study was very low. However, a bias cannot be ruled out as primers targeting specifically NRPS systems from actinomycetes were used [25]. If primer sets targeting different groups of microorganisms were employed, a higher diversity of NRPS genes would be expected. The fact that a closely related NRPS sequence clade was found in all sponge samples independent of their geographic location, in Caribbean seawater and in a Mediterranean streptomycete isolate suggests a wide geographic distribution of this NRPS-bearing bacterium. 

### 2.2. NRPS-Containing Metagenomic Cosmid Clone

**Figure 2:**
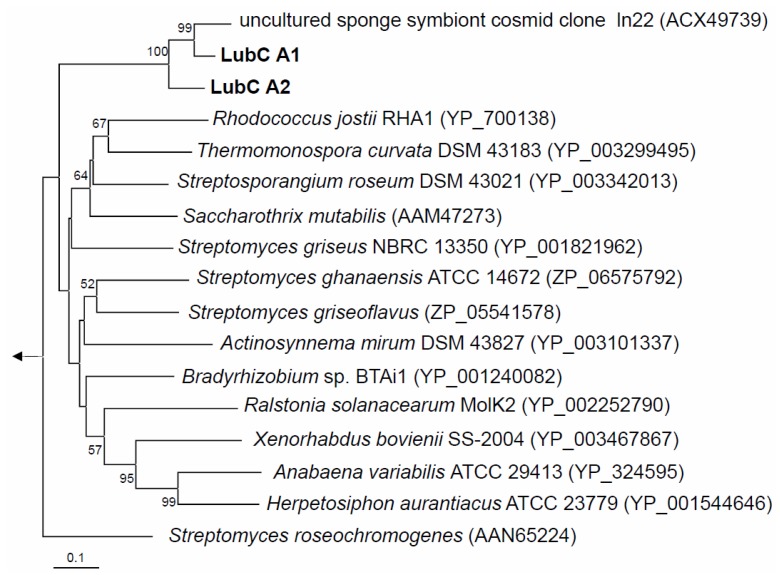
Neighbor-joining tree of translated NRPS A domains. Bootstrap values greater than 50% are indicated at the nodes. LubC A1 and A2 are the two adenylation domains from the NRPS structural gene *lubC*, which contains the two modules each bearing a single adenylation domain (see Figure 3). The arrow points to the archaeal outgroup, *Methanothermobacter thermoautotrophicus* (NP_275799). The scale bar indicates 0.1 substitutions per amino acid position.

PCR screening using degenerate primers [25] targeting two independently constructed metagenomic libraries harboring altogether *ca*. 2.4 Gb *Aplysina aerophoba* microbial community DNA [22] resulted in the identification of 14 NRPS-positive library pools, that is, at least 14 NRPS-containing cosmid clones. Sequence analysis of the NRPS PCR products from AApAY1 [22] NRPS-positive, metagenomic cosmid clones revealed nearly identical DNA sequences (data not shown). Two overlapping cosmid clones were sequenced and assembled into a 57031 bp long DNA fragment (AANRPS; Acc# HQ456128) with an overall GC content of 67%. Remarkably, the entire DNA fragment did not exhibit significant similarity on the DNA level to any known sequence in the database, except for NRPS identified previously in the *Chloroflexi* symbiont of *A. aerophoba* [21]. Phylogenetic analysis of the translated NRPS A domains confirmed the NRPS gene of a *Chloroflexi* symbiont of *A. aerophoba* (“uncultured sponge symbiont cosmid clone ln22” (ACX49739)) as the nearest phylogenetic relative (Figure 2) [21]. Those two NRPS genes share high protein identity (82%) and high similarity (87%) on the DNA level. Beside the NRPS gene and the efflux protein (lubB), there are however no further similarities in the gene neighborhood between the two cosmid clones.

**Figure 3:**
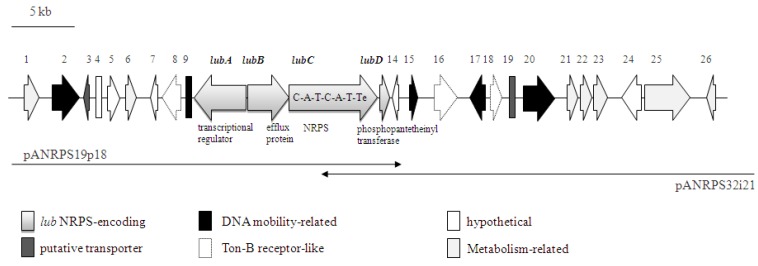
Genetic organization of the NRPS containing metagenomic cosmid clone from *Aplysina aerophoba* (AANRPS; Acc# HQ456128). Overlapping cosmids pANRPS19p18 and pANRPS32i21 and ORF numbers are indicated. Putative functions of *lub* genes-encoded NRPS proteins, as well as domain organization of *lubC* are depicted.

The genomic organization of the NRPS-containing cosmid clone is shown in Figure 3. At least 26 putative ORFs were identified (Table 1). Of these, four ORFs are proposed to represent the gene cluster coding for NRPS related proteins, which were termed *lubA*, *B*, *C*, *D*. The proposed gene cluster consists of 15752 bp and has a G + C content of 68%. The gene *lubA* encodes for a putative transcriptional regulator of NRPS expression, and is located upstream of the putative efflux protein-encoding gene *lubB*. The NRPS structural gene *lubC* contains two complete NRPS modules and is therefore predicted to encode biosynthesis of the dipeptide [28]. Both A1 and A2 adenylation domains probably use aromatic amino acids, such as phenylalanine and tyrosine, as substrates (NRPSpredictor2; [26]). Furthermore, three TonB-dependent receptors and a phosphopantetheinyl transferase (*lubD*) are contained on the metagenomic cosmid fragment (Table 1). TonB-dependent transporters are frequently involved in iron uptake via siderophores, as well as other substrates including heme, vitamin B_12_, proteins or polysaccharides [29]. The phosphopantetheinyl transferase enzyme (PPT) activates a carrier protein by the transfer of a phosphopantetheinyl moiety to a serine residue.

The postulated chemical product of this novel NRPS gene cluster remains elusive, owing to a lack of related NRPS genes in the databases and the lack of robust prediction tools. However, it has become clear that NRPS gene clusters are widespread in actinomycete strain collections with recoveries of NRPS genes from more than half of the isolates screened [18,19] as well as being abundant in microbial genomes/metagenomes. With ever cheaper sequencing technologies and improved bioinformatic prediction tools, genomic mining approaches will undoubtedly be instrumental for the identification of sources suitable for natural product discovery.

**Table 1:** Putative genes identified on the genomic fragment AANRPS (pANRPS19p18 and pANRPS32i21) from *Aplysina aerophoba*.

CDs	Position (nd)	Putative Function	Most Similar Homolog, (Acc#), origin	Identity/Similarity (%)					No. of Amino Acids
ORF1	3626-4807	Serine-threonine phosphatase	PrpA (NP_487771), *Nostoc* sp. PCC 7120	53/69					399
ORF2	5909-8170	Helicase RecD/TraA	BAL199_00820, (ZP_02192076), Alpha proteobacterium BAL199	72/82					753
ORF3	8441-8953	ABC transporter	Cagg_3718, (YP_002464990), *Chloroflexus aggregans* DSM 9485	42/51					170
ORF4	9894-10364	Hypothetical protein	MC7420_1635, (ZP_05030609), *Microcoleus chthonoplastes* PCC 7420	40/51					156
ORF5	10853-11857	Hypothetical protein	AM1_4437, (YP_001518731), *Acaryochloris marina* MBIC11017	34/52					334
ORF6	11903-12802	Hydrolase	VEx25_1601, (ZP_04922735), *Vibrio* sp. Ex25	29/41					299
ORF7	13907-14512	Hypothetical protein	BACCOPRO_03255, (ZP_03644864), *Bacteroides coprophilus* DSM 18228	26/40					201
ORF8	14867-16432	TonB-dependent receptor	MXAN_6044, (YP_634179), *Myxococcus xanthus* DK 1622	35/54					521
ORF9	16943-17452	Exonuclease	RLO149_22990, (ZP_02142576), *Roseobacter litoralis* Och 149	39/51					169
lubA	17745-22022	LuxR transcriptional regulator	HNE_2502, (YP_761196), *Hyphomonas neptunium* ATCC 15444	26/44	1429
lubB	21850-25236	Resistance protein	Sputw3181_3288, (YP_964656), *Shewanella* sp. W3-18-1	43/63	1128
lubC	25260-32465	NRPS (C-A-T-C-A-T-Te)	Siderophore, (ACX49739), uncultured marine bacterium 1n22	81/87	2401
lubD	32655-33497	Phosphopantetheinyl transferase	Mnod_1716, (YP_002497009), *Methylobacterium nodulans* ORS 2060	36/46	280
ORF14	33681-34217	Hypothetical protein	MldDRAFT_3697, (ZP_01290808), Delta proteobacterium MLMS-1	63/77	178
ORF15	35067-35822	Transposase	EbA6749, (YP_160886), *Aromatoleum aromaticum* EbN1	58/74	251
ORF16	37148-38974	TonB-dependent receptor	Sama_2896, (YP_928768), *Shewanella amazonensis* SB2B	65/82	608
ORF17	40041-41315	Transposase	BAL199_06759, (ZP_02191799), Alpha proteobacterium BAL199	45/56	424
ORF18	41701-42630	TonB-dependent receptor	GPB2148_3348, (ZP_05093557), marine Gamma proteobacterium HTCC2148	44/59	309
ORF19	43924-44364	Membrane transport protein	Ykris0001_15620, (ZP_04625017), *Yersinia kristensenii* ATCC 33638	43/56	146
ORF20	44366-46975	DNA invertase	NB231_12409, (ZP_01126794), *Nitrococcus mobilis* Nb-231	66/78	869
ORF21	47982-48890	Methyltransferase	MaviaA2_010100001311, (ZP_05214826), *Mycobacterium avium* ATCC 25291	40/49	302
ORF22	49061-50023	NADH-quinone oxidoreductase	Psta_3148, (YP_003371672), *Pirellula staleyi* DSM 6068	25/40	320
ORF23	50133-51254	2,3-Dihydroxybenzoic acid decarboxylase	PJE062_2683, (ZP_05084178), *Pseudovibrio* sp. JE062	65/75	373
ORF24	52396-54060	Hypothetical protein	ZP_05710821, (ZP_05710821), *Desulfurivibrio alkaliphilus* AHT2	49/68	554
ORF25	54272-58006	Cyclopropane-fatty-acyl-phospholipid synthase	ADG881_908, (ZP_05041385), *Alcanivorax* sp. DG881	51/66	1244
ORF26	59373-60101	Nucleoside 2-deoxyribosyltransferase	P9211_14861, (YP_001551371), *Prochlorococcus marinus* str. MIT 9211	63/76	242

## 3. Experimental Section

### 3.1. Sponge Collection

Marine sponges were collected by SCUBA diving at a depth of 5 to 15 m: *Aplysina aerophoba* offshore from Banyuls sur mer, France (GPS: 42°29′N, 03°08′E); *Agelas citrina*, *Aplysina archeri*, *Aplysina cauliformis*, *Aplysina insularis*, *Callyspongia vaginalis*, *Niphates digitalis*, *Plakortis* sp., *Smenospongia aurea*, *Tedania ignis*, *Verongula gigantea*, *Xestospongia muta* offshore from Patch Reef, Bahamas (GPS: 24°14′N, 74°32′W). Additionally, the ascidian *Ecteinascidia turbinata*, sediment and seawater samples were collected from the sampling site at Patch Reef, Bahamas. Individual specimens were placed separately in plastic bags and brought to the surface. The sponge and ascidian tissues were cut into pieces and stored at −80 °C until use. 

### 3.2. Cultivation and Identification of Sponge-Associated Bacteria

Strain *Streptomyces* sp. Aer003 was cultivated from the sponge *Aplysina aerophoba* using M1 [30] culture medium and identified by 16S rRNA gene sequencing as described previously by Hentschel *et al*. [31]. 

### 3.3. DNA Extraction, PCR Amplification and Sequencing of A Domains of NRPS Genes

Genomic DNA was isolated from freshly collected sponges, ascidian and seawater following the method as described previously by Fieseler *et al*. [22] using the FAST DNA Spin kit for Soil (Q-Biogene). Amplification of the A domains of NRPS gene fragments was performed as described previously [25] using degenerate primers A7R (5′-SASGTCVCCSGTSCGGTAS-3′) and A3 (5′-GCSTACSYSATSTACACSTCSGG-3′). PCR amplification products of *ca*. 750 bp in size were cloned into a pGEM-Teasy vector (Promega) and transformed into electrocompetent *Escherichia coli* XL1-Blue cells. Plasmid minipreps by alkaline lysis procedures and sequencing of the inserts were performed as described previously [22]. The same protocol was followed for the strain *Streptomyces* sp. Aer003.

### 3.4. Metagenomic Library Construction and Screening for NRPS-Encoding Clones

Two metagenomic libraries constructed from microbial cells of the marine sponge *Aplysina aerophoba* [22] using an *E. coli*-*Streptomyces* shuttle cosmid vector, pAY1, [32] were used for NRPS screening. The libraries represented a total of *ca*. 2.4 Gb of sponge-associated microbial DNA. Library pools were screened by PCR following the method of Piel *et al*. [33] using the degenerate primers targeting the A domains of NRPS genes (A7R and A3; see sequences above). Two PCR-positive overlapping cosmid clones were sequenced (pANRPS19p18 and pANRPS32i21). 

### 3.5. Sequence Analysis

Sequencing analysis was performed by Agowa/LGC Genomics, Berlin, Germany. Sequence data were assembled and annotated using the Vector NTI software (Invitrogen) and analyzed using EMBOSS-Transeq and BLAST algorithms [34]. 

## 4. Conclusions

Genomic mining revealed the wide distribution of a single NRPS module that is phylogenetically related to actinomycetes and for which hydroxy-phenyl-glycine is the predicted substrate. This NRPS module has been identified in 12 marine sponge species from disparate geographic locations, in one ascidian, an actinobacterial isolate and in seawater. Two additional NRPS gene sequences with valine as predicted substrate were also identified. Metagenomic approaches furthermore revealed a phylogenetically different NRPS gene cluster that has previously been appointed to a *Chloroflexi* sponge symbiont. The chemical nature and putative bioactivity of the postulated NRPS as well as its possible role in the symbiosis context remain to be explored in future studies. The implementation of metagenomic approaches, such as presented here, are beginning to shed glimpses of light on the secondary metabolite biosynthesis gene repertoire of sponge symbionts, which are still inaccessible by conventional cultivation techniques. 
